# Detection and Quantification of Viable but Non-culturable *Campylobacter jejuni*

**DOI:** 10.3389/fmicb.2019.02920

**Published:** 2020-01-10

**Authors:** Ruiling Lv, Kaidi Wang, Jinsong Feng, Dustin D. Heeney, Donghong Liu, Xiaonan Lu

**Affiliations:** ^1^Food, Nutrition, and Health Program, Faculty of Land and Food Systems, The University of British Columbia, Vancouver, BC, Canada; ^2^College of Biosystems Engineering and Food Science, Zhejiang University, Hangzhou, China

**Keywords:** *Campylobacter*, food safety, viable but non-culturable, quantitative PCR, intercalating agent

## Abstract

*Campylobacter* can enter a viable but non-culturable (VBNC) state to evade various stresses, and this state is undetectable using traditional microbiological culturing techniques. These VBNC bacterial cells retain metabolism and demonstrate pathogenic potential due to their ability to resuscitate under favorable conditions. Rapid and accurate determination of VBNC *Campylobacter* is critical to further understand the induction and resuscitation of the dormancy state of this microbe in the agri-food system. Here, we integrated propidium monoazide (PMA) with real-time polymerase chain reaction (qPCR) targeting the *rpoB* gene to detect and quantify *Campylobacter jejuni* in the VBNC state. First, we optimized the concentration of PMA (20 μM) that could significantly inhibit the amplification of dead cells by qPCR with no significant interference on the amplification of viable cell DNA. PMA-qPCR was highly specific to *C. jejuni* with a limit of detection (LOD) of 2.43 log CFU/ml in pure bacterial culture. A standard curve for *C. jejuni* cell concentrations was established with the correlation coefficient of 0.9999 at the linear range of 3.43 to 8.43 log CFU/ml. Induction of *C. jejuni* into the VBNC state by osmotic stress (i.e., 7% NaCl) was rapid (<48 h) and effective (>10% population). The LOD of PMA-qPCR for VBNC *C. jejuni* exogenously applied to chicken breasts was 3.12 log CFU/g. In conclusion, PMA-qPCR is a rapid, specific, and sensitive method for the detection and quantification of VBNC *C. jejuni* in poultry products. This technique can give insight into the prevalence of VBNC *Campylobacter* in the environment and agri-food production system.

## Introduction

*Campylobacter* is responsible for the most frequently reported foodborne gastrointestinal infection in the world (Silva et al., 2011). It is a microaerobic bacterium but highly prevalent in the aerobic food processing environment, such as poultry farms and slaughter facilities. The Centers for Disease Control indicates that *Campylobacter* spp. caused a total of 472 foodborne outbreaks, 4,209 illnesses, and 315 hospitalizations from 2011 to 2017 in the United States (CDC, 2018). Among them, *Campylobacter jejuni* is the major species that represent 95% of the total contaminations. Typical transmission routes include contaminated dairy, water, and poultry products, with poultry products associated with 25% of the cases (Silva et al., 2011).

*Campylobacter jejuni* can enter a viable but non-culturable (VBNC) state upon exposure to various stress, including low temperature, oxygen, acid treatment, and salt treatment (Silva et al., 2011). VBNC cells are unable to divide in the conventional culture media while they retain membrane integrity and metabolic activity (Ramamurthy et al., 2014). The VBNC state is considered by some researchers to be related to other stress-induced phenotypes, such as antibiotic-tolerant persister cells, and is hypothesized to be a terminal stage in the dormancy continuum (Oliver, 2005). To date, 85 bacterial species have been described to form VBNC cells under stress conditions, including *Escherichia*, *Vibrio*, *Listeria*, and *Campylobacter* (Pinto et al., 2015). Bacterial cells in the VBNC stage can remain dormant for several months before resuscitation under favorable conditions (Baffone et al., 2006). For example, VBNC *C. jejuni* cells have been reported to resuscitate *in vivo* with mouse infections (Baffone et al., 2006), in microaerobic conditions (Bovill and Mackey, 1997), and in embryonated chicken eggs (Cappelier et al., 1999). VBNC cells are thought to be avirulent due to a reduced rate of gene expression and protein translation required for pathogenesis. However, VBNC cells that become resuscitated can regain full infective phenotypes (Baffone et al., 2006; Pinto et al., 2015), representing a real threat to the public health.

Current methods for the detection of *C. jejuni* are widely culture dependent, which severely underestimate the presence of VBNC cells (Baffone et al., 2006). Several assays have been applied to the detection of VBNC cells, such as the direct fluorescent antibody–direct viable count (DFA–DVC) method, substrate responsiveness combined with fluorescent *in situ* hybridization (DVC–FISH assay), and LIVE/DEAD BacLight bacteria viability kit combined with flow cytometry. However, most of these methods are costly, unspecific, technically challenging, or unable to conduct quantification. Therefore, it is imperative that we develop novel methods for the detection and quantification of VBNC *C. jejuni* in the agri-food system. To this point, molecular techniques have been developed to detect and identify *C. jejuni* in the environment, especially with DNA amplification methods, including polymerase chain reaction (PCR) and variations thereof (Magajna and Schraft, 2015; Castro et al., 2018). Critically, PCR and quantitative PCR (qPCR) methods are not able to differentiate between viable and non-viable (dead) bacterial cells. One promising method for the detection of viable cells in a mixture of live and dead cells is the use of propidium monoazide (PMA) coupled to qPCR (Magajna and Schraft, 2015; Castro et al., 2018). PMA creates strong covalent bonds to double-stranded DNA after photoactivation, but the bulky structure of this molecule and the positive charges prevent itself from entering bacterial cells with intact membranes (Nocker et al., 2006). PMA-bound DNA inhibits DNA polymerases and therefore is not amplified during reactions, enabling the differentiation between viable and dead cells. Thus, VBNC cell count can be estimated by subtracting the number of culturable cells from the total viable cell count determined using PMA-qPCR. This technique has been used for the detection of different VBNC bacterial cells, such as *Escherichia coli* and *Vibrio parahaemolyticus* (Afari and Hung, 2018; Chang and Lin, 2018; Zhong and Zhao, 2018; Telli and Dogruer, 2019).

In the current study, we implemented and optimized the PMA-qPCR method to quantify viable cells in pure cultures of *C. jejuni* in the background of dead cells. VBNC cells of *C. jejuni* were then induced by osmotic stress and artificially contaminated onto commercial chicken breasts to validate the performance of PMA-qPCR coupled with the plating assay.

## Materials and Methods

### Bacterial Strains and Growth Conditions

Bacterial strains are listed in Table 1. *C. jejuni* was routinely cultivated under microaerobic conditions (85% N_2_, 10% CO_2_, and 5% O_2_) on Mueller–Hinton (MH) agar (BD Difco, Thermo Fisher Scientific, Waltham, United States) supplemented with 5% defibrinated sheep blood (Alere Inc., Stittsville, ON, Canada) at 37°C. *C. jejuni* broth cultures were prepared by picking a single bacterial colony into the MH broth (BD Difco, Thermo Fisher Scientific, Waltham, United States) with constant shaking at 175 rpm for 16–18 h at the same aforementioned conditions.

**TABLE 1:** Bacterial strains used for the specificity test of qPCR.

**Bacterial species**	**Strain**	**Source**	**PMA-qPCR result**
*Campylobacter jejuni*	ATCC 33560	Bovine feces	+
	F38011	Human clinical isolate	+
	1658	Human clinical isolate	+
	NCTC 11168	Human clinical isolate	+
	81–116	Human clinical isolate	+
*Campylobacter coli*	RM 1875	Human clinical isolate	
	RM 2228	Human clinical isolate	-
	RM 5611	Human clinical isolate	-
*Escherichia coli*	O103:H2	Bovine feces	-
	O118:H16	Bovine feces	-
*Salmonella enteritis*	0EA2699	Human clinical isolate	-
	3512H	Human clinical isolate	-
*Listeria monocytogenes*	SEA 15B88	Human clinical isolate	-
*Listeria monocytogenes*	15B98	Human clinical isolate	-
*Pseudomonas aeruginosa*	H288	Human clinical isolate	-

### PMA Treatment and DNA Extraction

Propidium monoazide (Biotium, Fremont, United States) was diluted and added to 450 μl of unwashed bacterial cell culture at various concentrations into 1.7-ml graduated micro-centrifuge tubes manufactured from resin (LifeGene, Modiín, Israel). A bacterial cell–PMA mixture was kept in the dark on ice with constant shaking at 150 rpm for 10 min. Then, DNA crosslinking was performed by exposing the tubes horizontally to a 300-W halogen light (120 V; GE Lighting, General Electric Co., Cleveland, United States) at a distance of 20 cm for 10 min. The mixture was centrifuged at 15,000 × *g* and washed one time with sterile distilled deionized water (ddH_2_O) to remove the residual PMA before DNA extraction. Genomic DNA of pure bacterial cultures was extracted by thermal treatment at 100°C for 10 min, followed by incubation on ice for 10 min. To compare the extraction efficiency, DNA extraction of both pure bacterial cultures and spiked chicken carcasses was also performed using the Presto Mini gDNA Bacteria Kit (Geneaid, Taiwan, China) as specified by the manufacturer. The quality and concentration of DNA were determined using a NanoDrop UV-Vis spectrophotometer (Thermo Fisher Scientific, Waltham, United States). DNA was stored at -20°C until qPCR analysis. The optimal concentration of PMA required for further qPCR experiments was determined using live and heat-inactivated *C. jejuni* cells. Live cells at different concentrations were collected by diluting the exponential phase culture, and dead cells were prepared by heating 1 ml of 6 log CFU/ml *C. jejuni* culture at 90°C for 5 min in 1.7-ml micro-centrifuge tubes as aforementioned using a heat block (VWR International, Pennsylvania, United States) (Pacholewicz et al., 2013). The death rate was confirmed by the plating assay (data not shown). Separate samples containing 6 log CFU/ml of live and heat-inactivated *C. jejuni* cells were treated with PMA at concentrations of 0, 10, 15, 20, 50, and 100 μM, respectively, prior to DNA extraction as previously described.

### PMA-qPCR

The primers targeting the DNA-directed RNA polymerase *rpoB* were used for *C. jejuni* detection as previously described (Silvestri et al., 2017). The sequences of the primers were *rpoB* 1 (5′-GAGTAAGCTTGCTAAGATTAAAG-3′) and *rpoB* 2 (5′-AAGAAGTTTTAGAGTTTCTCC-3′), and the amplicon length was 121 bp. Each qPCR reaction (total 20 μl) contained 1 × SensiFAST SYBR Mix (Bioline, Taunton, United States), 2 μl of DNA temple, 100 nM of each primer, and sterile ddH_2_O. The qPCRs were performed in an Applied Biosystems 7500 Real Time PCR system (Thermo Fisher Scientific, Waltham, MA, United States) with an initial denaturation at 50°C for 2 min and 95°C for 10 min, followed by 40 cycles of 95°C for 15 s and 60°C for 1 min. A negative control (ddH_2_O) was included in each qPCR run, and each sample was tested in triplicate. The specificity of the *rpoB* primer set was evaluated using 15 different bacterial strains as listed in Table 1 in the optimized PMA-qPCR assay. The sensitivity of the PMA-qPCR assay was determined with 10-fold serial dilutions of viable *C. jejuni* F38011 cells ranging from 1 to 8 log CFU/ml in a background of 6 log CFU/ml dead cells (inactivated at 90°C). The lowest CFU/ml of *C. jejuni* that generated Ct values < 35 was considered as the limit of detection (LOD) of this assay. A standard curve correlating Ct value and known concentration of *C. jejuni* cells was established and used to estimate the amplification efficiency and quantification capability. The amplification efficiency (*E*) was calculated using the slope of the standard curve *E* = [10^(–1/slope)^ − 1] ^∗^ 100%.

### Induction of VBNC *C. jejuni*

Overnight *C. jejuni* culture was adjusted to OD_600_ = 0.3 (∼9 log CFU/ml) and washed with phosphate-buffered saline (PBS). Bacterial cells were then suspended in 7% (w/v) NaCl solution to a final concentration of 8 log CFU/ml and incubated for 48 h at 37°C. The culturable cell population and viable cell population were separately monitored by the plating assay and PMA-qPCR at 3, 6, 9, 12, 24, and 48 h. VBNC cell concentrations were estimated as the difference between culturable cells and viable cells. The LOD of the plating assay was determined to be 1 CFU/ml based on the presence of one colony on the MH blood agar with 1 ml of bacterial sample (Silvestri et al., 2017). When the culturable cell concentration was less than 1 CFU/ml, it was considered that all of the viable cells quantified by PMA-qPCR were VBNC cells. As validation, 100 μl of the putative VBNC cell suspensions was transferred into the fresh MH broth and incubated under the optimum microaerobic conditions for 72 h to confirm that no cells could be resuscitated.

### Detection of VBNC *C. jejuni* in Chicken Samples

The reliability of PMA-qPCR was investigated by the detection of VBNC *C. jejuni* in chicken samples. Boneless and skinless chicken breasts were purchased from a local supermarket in Vancouver and stored at −20°C. Chicken was cut to ∼25 g per piece and exposed to 250 ml of 1% (w/v) chlorine for 10 min, followed by three times of washing with ddH_2_O before drying for 1 h at 22°C. The chicken sample was contaminated with 100 μl of VBNC cells induced by osmotic stress for 48 h at the final concentrations ranging from 1.12 to 7.12 log CFU/ml and left to air-dry at 22°C for 1 h.

To recover the VBNC cells for determination, inoculated chicken samples were separately placed in the sterile sample bags and rinsed with 10 ml of peptone water. After manually massaging the bag for 3 min at 22°C, 1 ml of liquid was collected for DNA extraction and PMA-qPCR. A standard curve was generated by comparing Ct values to the known concentrations of VBNC cells applied to the chicken samples. Spiking of ddH_2_O onto chicken samples was regarded as the negative control. Recovery of non-treated culturable *Campylobacter* with the aforementioned rinsing procedure was assessed by the plating assay.

### Statistical Analysis

All the trials were conducted with at least three replicates in each experiment. Means and standard deviations of Ct values were calculated using the Microsoft Excel program (Seattle, United States). Figures were generated using the Origin software (Version 9.2, Origin Corp., Boston, MA, United States). One-way analysis of variance (ANOVA), followed by Duncan’s test for multiple comparisons with a confidence level of 95% (*P* < 0.05), was performed using the SPSS Statistics software (Version 20, IBM, United States).

## Results and Discussion

*Campylobacter* is the leading cause of bacterial diarrheal disease worldwide (Burnham and Hendrixson, 2018). Due to the wide consumption and high prevalence of *C. jejuni* in poultry, this food source is considered as the major transmission route for *C. jejuni* infections (Silva et al., 2011). Unfavorable environmental conditions, such as desiccation, aerobic stress, and starvation, can induce *C. jejuni* to enter the VBNC state and evade detection using the culture-based methods (Baffone et al., 2006). These VBNC cells can remain viable for an extended time period, resuscitate under favorable conditions, recover metabolism and virulence (Ramamurthy et al., 2014; Pinto et al., 2015), and subsequently pose an important concern to the public health. Few research has been performed on the detection and quantification of VBNC *C. jejuni* yet.

### Optimization of PMA Concentration

VBNC cells are now recognized to coexist with culturable and dead cells in bacterial cultures grown in the standardized laboratory condition (Kassem et al., 2013; Bronowski et al., 2014; Ayrapetyan et al., 2018). Therefore, we used a DNA-intercalating dye (i.e., PMA) to inhibit the amplification of DNA present in the dead cells. Previous studies identified 2–100 μM of PMA to be the optimal concentration range that was specific and compatible to determine the viability of each bacterial species in different matrices (Magajna and Schraft, 2015; Castro et al., 2018; Salas-Masso et al., 2019). PMA treatment with a higher concentration could inhibit DNA amplification of viable cells and lead to underestimation of viable cell count or even false-negative results. On the contrary, a lower concentration of PMA may not be fully effective to inhibit the signal from the dead cells and may cause overevaluation. Thus, it is crucial to determine the optimal concentration of PMA as the pretreatment for assay performance.

**FIGURE 1:**
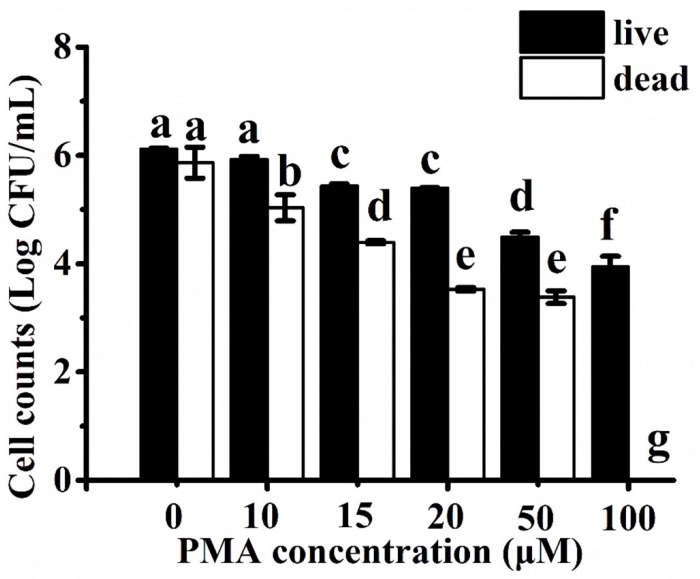
*Campylobacter jejuni* cell counts estimated using Ct values from the propidium monoazide (PMA) with real-time polymerase chain reaction (qPCR) assay after treatment with different concentrations of PMA. Live and heat-inactivated cells at 6 log CFU/ml were treated and tested separately. Different letters indicated significant differences among different groups (*P* < 0.05).

To compare the ability of PMA to differentiate between viable and heat-inactivated *C. jejuni* cells, each culture was separately treated with increasing concentrations of PMA (i.e., 0–100 μM) before DNA extraction and qPCR. DNA amplification curves from 6 log CFU/ml of viable cells treated with PMA at concentrations of <20 μM were similar to the non-PMA-treated samples (<1 log CFU/ml reduction). DNA amplification of live cells was significantly inhibited (>2 log CFU/ml reduction) when treated with concentrations of PMA above 20 μM (Figure 1). In contrast, the amplification of DNA from 6 log CFU/ml of dead cells decreased in a PMA dose-dependent manner up to 20 μM (Figure 1). There was no significant difference (*P* > 0.05) in the amplification when the dead cells were treated with 20 or 50 μM of PMA, while no amplification from the dead cells was observed at 100 μM of PMA. Therefore, 20 μM of PMA was used in the following study as it could effectively inhibit DNA amplification of the dead *C. jejuni* cells without significant influence on quantifying viable bacterial cells.

Previous studies indicated that PMA-qPCR was an effective approach to quantify viable *Campylobacter* cells in different sample matrices (Josefsen et al., 2010; Melero et al., 2011; Seinige et al., 2014). However, the optimal concentration of PMA varied among different studies. For example, Josefsen et al. (2010) reported that PMA treatment of 10 μg/ml (23.81 μM) achieved complete inhibition of the qPCR signal from the dead *Campylobacter* cells at a concentration of 6 log CFU/ml, but Pacholewicz et al. (2013) failed to completely inhibit the signal of dead cells >4 log CFU/ml by using a similar PMA-qPCR approach. In other studies, 47 and 50 μM of PMA were separately used to enumerate viable *Campylobacter* in chicken meat (Seinige et al., 2014; Castro et al., 2018). The variations in the concentration of PMA applied in the aforementioned studies might be due to the diverse sensitivity of the cell membrane of different *Campylobacter* strains as well as the penetration power of PMA to the corresponding viable and dead cells (Fittipaldi et al., 2012). Other variables were also introduced into these studies, including the amplicon size, sample matrices, the power and type of light, incubation time, and photoactivation time (Pacholewicz et al., 2013). In addition, other DNA-intercalating dyes, such as ethidium monoazide (EMA), have been used to couple with qPCR for the quantification of viable *Campylobacter* cells (Webb et al., 2016). PMA was selected in the current study because it has a lower cytotoxicity and a wider range of application for both Gram-negative and Gram-positive cells than EMA (Nocker and Camper, 2009; Fittipaldi et al., 2012).

### Specificity and Sensitivity of PMA-qPCR Assay for *C. jejuni* Pure Culture

The specificity of the *rpoB* primer set was determined by testing DNA from a total of 15 strains from six different bacterial species (Table 1). The application of DNA from six *C. jejuni* strains all resulted in positive amplification (Ct value ranging from 20.614 to 24.926). In comparison, no amplification was observed from the genomic DNA extracted from other bacterial species. We therefore validated 100% specificity for the detection of *C. jejuni*. The *rpoB* gene was also targeted in a previous study of using qPCR for the detection of viable *C. jejuni* due to its high sensitivity, heterogeneity, and suitable size of the amplification product (Silvestri et al., 2017).

**FIGURE 2:**
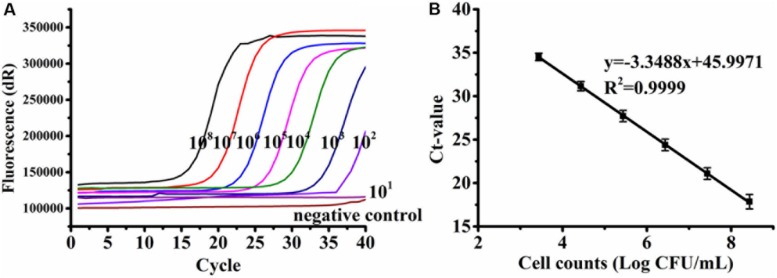
Representative amplification curves **(A)** and standard curve **(B)** generated from 10-fold serial dilutions of viable *Campylobacter jejuni* F38011 cells ranging from 2.43 to 8.43 log CFU/ml in the background of 6 log CFU/ml of dead cells. Bacterial genomic DNA was extracted using the boiling method. Standard deviations were calculated based upon three replicates.

A standard curve was established from the serially diluted viable *C. jejuni* F38011 cells in the background of 6 log CFU/ml of dead cells in relation to the Ct values. The linear regression slope was determined to be −3.3379 with a correlation coefficient (*R*^2^) of 0.9999 (Figure 2), and PCR amplification efficiency was calculated to be 99.34%. The standard curve was linear over a range of 3.43 to 8.43 log CFU/ml, and the LOD of PMA-qPCR was determined to be 2.43 log CFU/ml. Other qPCR assays developed in the previous studies reported LODs for *Campylobacter* to be 1 log CFU/ml (Melero et al., 2011), 1.5 log CFU/ml (Tsiouris et al., 2019), and 2 log CFU/ml (Josefsen et al., 2010). Several variables including the quantification of total cells (dead and viable) versus viable cells, the use of different intercalating dyes (e.g., EMA), bacterial strains, designed primers, DNA extraction protocol, and qPCR procedure could contribute to the difference of the reported LODs.

Various DNA extraction methods may influence the sensitivity and effectiveness of qPCR due to differences in quantity and/or quality of the extracted nucleic acids (Demeke et al., 2009). Here, similar standard curves were developed when genomic DNA was extracted from *C. jejuni* F38011 using either a cheaper boiling method (Figure 2B) or a more expensive commercial extraction kit (Supplementary Figure S1), demonstrating that the amplification efficiency of qPCR was consistent between the two extraction methods. PMA-qPCR was applied to separately amplify the DNA extracted from the pure culture of four *C. jejuni* strains and establish the corresponding standard curves. The correlation coefficients (*R*^2^) of all the four standard curves ranged from 0.9993 to 1 (Supplementary Figure S1), indicating that the developed PMA-qPCR assay was accurate and capable of quantifying various *C. jejuni* strains with a dynamic concentration range of 3 to 8 log CFU/ml.

### Induction of VBNC *C. jejuni* by Osmotic Stress

**FIGURE 3:**
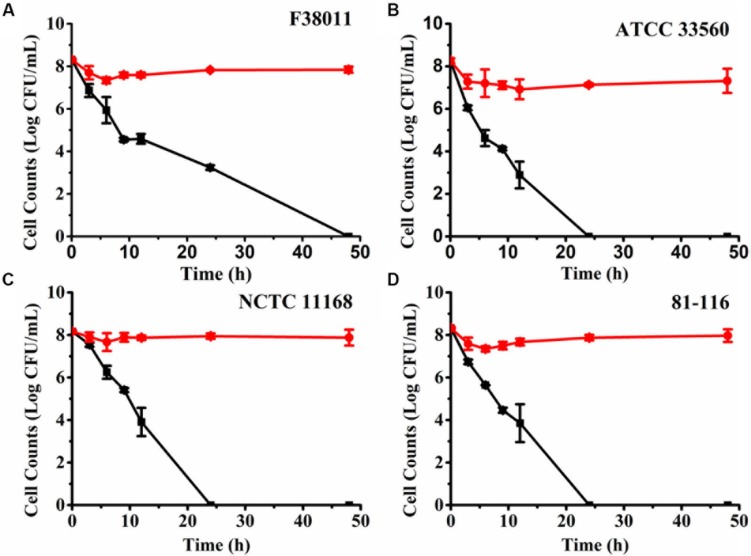
Induction of viable but non-culturable (VBNC) *Campylobacter jejuni* F38011 **(A)**, ATCC 33560 **(B)**, NCTC 11168 **(C)** and 81-116 **(D)** under osmotic pressure in 7% (w/v) NaCl solution. Red circles (⚫) represent the viable cell counts quantified using PMA with real-time polymerase chain reaction (qPCR), while black squares (■) represent culturable cell counts determined using the plating assay. The difference between viable cells and culturable cells was considered as VBNC bacterial cells. The error bar was calculated based upon three replicates.

Osmotic stress is an effective and rapid approach to induce bacteria into the VBNC state (Magajna and Schraft, 2015). NaCl is a potent antimicrobial that has been commonly used in food preservation (Csonka, 1989). Thus, 7% (w/v) NaCl solution was used to provide osmotic stress and induce *C. jejuni* into the VBNC state. The number of culturable and viable cells under osmotic stress was separately monitored using the plating assay and PMA-qPCR (Figure 3). For *C. jejuni* F38011, culturable cell counts decreased rapidly at the beginning of the osmotic treatment (3.76 log CFU/ml reduction at 37°C after 9 h) and continued over time, but the reduction of viable cell counts was negligible (<1 log CFU/ml) up to 48 h (Figure 3A). The difference between viable and culturable cell counts increased over time, indicating the accumulation of VBNC *C. jejuni* cells upon prolonged exposure to the osmotic stress. After osmotic treatment for 48 h, no culturable *C. jejuni* cells were detected by the plating assay (<1 CFU/ml), and no bacterial growth was observed after enrichment in MH broth for 72 h in a microaerobic condition at 37°C. *C. jejuni* F38011 had an estimated VBNC cell count of 7.84 log CFU/ml after 48 h of incubation with the osmotic stress.

When other *C. jejuni* strains were treated with the osmotic stress (Figures 3B–D), there was complete loss of culturability after an average of 24 h. In comparison, the viable cell counts were stagnant (average 7.98 log CFU/ml) over 48 h. The resistance to osmotic stress and progression into the VBNC state varied among different *C. jejuni* strains. In comparison, *E. coli* O157:H7 required 72 h of incubation in a 13% (w/v) NaCl solution to transit all viable cells into the VBNC state (Makino et al., 2000). The same transition was observed to require 5 days in *Salmonella enterica* in a 7% (w/v) NaCl solution (Kusumoto et al., 2012). Thus, *C. jejuni* demonstrated less tolerance to the osmotic stress than other enteric pathogens and exhibited a swifter progression into the VBNC state.

It is known that bacteria constantly sense and adapt to osmotic stress by the accumulation of intracellular ions (e.g., K^+^) and synthesis or import of compatible solutes so as to continue normal metabolism and cellular functions (McLaggan et al., 1994; Kempf and Bremer, 1998). For example, *E. coli* contains the Bet system and ProP/ProU transporter system to synthesize and transport glycine betaine in a high-osmolality environment (Jackson et al., 2009). However, *C. jejuni* lacks these typical osmotic protective systems that are common in other Gram-negative bacteria (Garenaux et al., 2008; Cameron et al., 2012). Instead, several genes have been implicated in responding to hyperosmotic conditions in *C. jejuni*, including *htrB* (encoding high-temperature response protein B), *ppk* (encoding polyphosphate kinase), a sensor histidine kinase (CJM1_1208) (Bronowski et al., 2017), and *gltD* and *glnA* that encode proteins for glutamate and glutamine synthesis, respectively (Cameron et al., 2012). Further study is required to determine if these genes are actively transcribed and play potential roles in the progression of *C. jejuni* into the VBNC state under osmotic stress or not.

### Quantification of VBNC *C. jejuni* in Poultry Products Using PMA-qPCR

**FIGURE 4:**
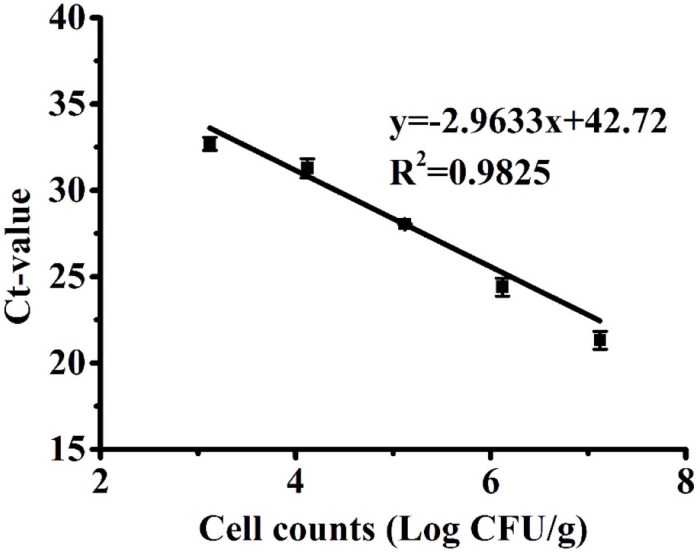
Standard curves produced from 10-fold serial dilutions of the VBNC cocktail of *Campylobacter jejuni* cells ranging from 3.12 to 7.12 log CFU/g recovered from chicken samples. The error bar was calculated based upon three replicates.

Since poultry is one of the major sources of *C. jejuni* contamination and a common vehicle for foodborne transmission of *Campylobacteriosis* (Young et al., 2007), we further evaluated the performance of PMA-qPCR to quantify the model contamination of chicken meat with VBNC *C. jejuni* cells. A VBNC bacterial cocktail of four *C. jejuni* strains induced by 7% (w/v) NaCl solution was spiked to chicken samples at known concentrations and subsequently determined using PMA-qPCR. The correlation coefficient (*R*^2^) between Ct values and the spiked concentration of VBNC *C. jejuni* cells was determined to be 0.9825, with a quantification range of 3.12 to 7.12 log CFU/g. There was no amplification observed in chicken samples that were not artificially contaminated, indicating that 1% (w/v) chlorine was an effective decontamination method used in the poultry industry to remove endogenous *C. jejuni* cells. In addition, the standard curve used for the chicken experiments (Figure 4) was established by first isolating VBNC cells and diluting them to known concentrations prior to application on the chicken samples. Considering that the efficiency of cell recovery and DNA extraction may vary among different initial concentrations of cells added to the chicken samples, it could explain why the amplification efficiency for this assay was relatively high (slope = −2.96, efficiency = 117%). The LOD of the PMA-qPCR assay for VBNC *C. jejuni* in chicken products was 3.12 log CFU/g (equivalent to 3.52 log CFU/ml of chicken rinse solution), which was higher than that determined in the pure bacterial culture (2.43 log CFU/ml), which was likely due to polymerase-inhibitory compounds in the chicken rinse solution, such as the background of dead bacterial cells and endogenous chicken molecules (Bae and Wuertz, 2012). It is also possible that the rinsing procedure could not fully recover all the VBNC cells spiked onto the chicken samples. In the preliminary spiking experiment, the recovery rate of culturable *C. jejuni* cells on chicken samples was 84.14%. The LOD of PMA-qPCR in the current study was improved in comparison to a previous study, in which qPCR exhibited a LOD of 1.2 × 10^4^ CFU/g for the quantification of viable *C. jejuni* in poultry neck-skin samples (Papic et al., 2017). Others employing PMA-qPCR to quantify *Campylobacter* in chicken carcasses reported LOD values as low as 10 CFU/g (Rantsiou et al., 2010) and 2 log CFU/ml (Josefsen et al., 2010). In other studies, PMA-qPCR was applied to detect VBNC *E. coli* O157:H7 in different agri-food products, with a LOD of 4.51 log CFU/ml in meatballs (Zhong and Zhao, 2018) and 3 log CFU/g in leaf (Dinu and Bach, 2013). The LODs derived from these studies were comparable to that in the current study. Variations in the gene/species targeted for amplification, primer design, PMA concentration, and DNA extraction method could account for some of these variations.

## Conclusion

A rapid, specific, and sensitive PMA-qPCR combined with the plating assay was developed for the detection and quantification VBNC *C. jejuni* in pure culture and poultry products. This method could be used to quantify the endogenous contamination of poultry and other food products by VBNC *C. jejuni* cells, which may evade detection by conventional culturing methods. The developed PMA-qPCR assay can assist in identifying sources of contamination and eventually reduce the prevalence of *C. jejuni* in the environment and agri-food system.

## Data Availability Statement

All datasets generated for this study are included in the article/Supplementary Material.

## Author Contributions

RL contributed to conducting bench work and manuscript writing. KW, JF, and DH contributed to the experimental design, conducting part of the bench work, and manuscript editing. DL contributed to the experimental design. XL contributed to the experimental design, and manuscript writing and editing.

## Conflict of Interest

The authors declare that the research was conducted in the absence of any commercial or financial relationships that could be construed as a potential conflict of interest.
