# Supplementary material for: Detection and Quantification of Viable but Non-culturable Campylobacter jejuni
Source: Front Microbiol. 2020 Jan 10;10:2920. doi: 10.3389/fmicb.2019.02920 (PMC6965164; doi:10.3389/fmicb.2019.02920)
Supplement: Supplementary file 1 [file Image_1.pdf]

Supplementary Information

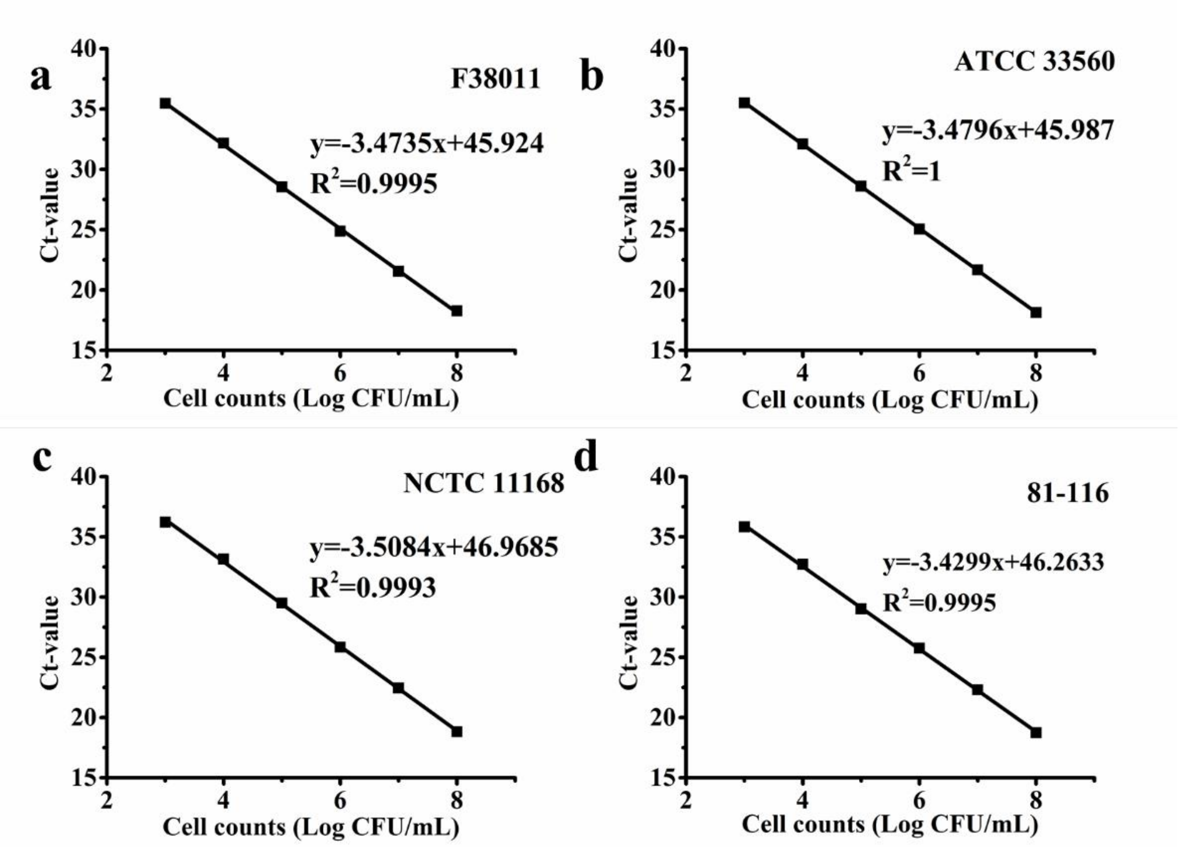

Figure S1. Standard curves produced from 10-fold serial dilutions of four different *C. jejuni* strains (a-d) ranging from 3 to 8 log CFU/mL. PMA-treated genomic DNA was extracted using the Presto Mini gDNA Bacteria Kit. Each data point represents three replicates.
